# Warfarin Pharmacogenomics: Designing Electrochemical DNA-Based Sensors to Detect CYP2C9*2 Gene Variation

**DOI:** 10.3390/genes16040372

**Published:** 2025-03-24

**Authors:** Tiago Barbosa, Stephanie L. Morais, Eduarda Pereira, Júlia M. C. S. Magalhães, Valentina F. Domingues, Hygor Ferreira-Fernandes, Giovanny Pinto, Marlene Santos, Maria Fátima Barroso

**Affiliations:** 1 REQUIMTE/LAQV, Escola Superior de Saúde, Instituto Politécnico do Porto, Rua Dr. António Bernardino de Almeida, 400, 4200-072 Porto, Portugal; tiagoesas2017@gmail.com (T.B.); mes@ess.ipp.pt (M.S.); 2 REQUIMTE/LAQV, Instituto Superior de Engenharia do Porto, Instituto Politécnico do Porto, Rua Dr. António Bernardino de Almeida, 431, 4249-015 Porto, Portugal; stephanielopesmorais@gmail.com (S.L.M.); vfd@isep.ipp.pt (V.F.D.); 3 REQUIMTE/LAQV, Departamento de Engenharia Química, Faculdade de Engenharia, Universidade do Porto, Rua Dr. Roberto Frias, 4200-465 Porto, Portugal; jmagalh@fe.up.pt; 4 Instituto de Educação, Ciência e Tecnologia Do Piauí (IFPI), Departamento de Informação, Ambiente, Saúde e Produção Alimentícia, 64019-368 Teresina, Brazil; hygorffernandes@ifpi.edu.br; 5 Grupo de Estudos Em Genética Humana e Médica (GEHMED), Laboratório de Genética e Biologia Molecular, Departamento de Biomedicina, Universidade do Delta do Parnaíba (UFDPar), 64202-020 Parnaíba, Brazil; giovanny@ufdpar.edu.br; 6 Grupo de Oncologia Molecular e Patologia Viral, Centro de Investigação, Instituto Português de Oncologia do Porto—Francisco Gentil, Rua Dr. António Bernardino de Almeida 865, 4200-072 Porto, Portugal

**Keywords:** cardiovascular diseases, CYP2C9*2, electrochemical DNA-based sensor, personalized medicine, pharmacogenetics, single-nucleotide polymorphisms

## Abstract

Background/Objectives: The CYP2C9 enzyme is involved in the metabolism of warfarin. The *CYP2C9* gene harbors several single-nucleotide polymorphisms (SNPs), including CYP2C9*2 (rs1799853), which is known to affect warfarin’s therapeutic response. So, it is important to develop analytical tools capable of genotyping these SNPs to adjust warfarin’s therapeutic outcomes. In this work, an electrochemical DNA-based sensor was constructed and optimized for the detection of the CYP2C9*2 polymorphism. Methods: Using bioinformatic database platforms, two 71 base pair DNA target probes with the polymorphic variants A and G were chosen and designed. A DNA-based sensor was composed by mercaptohexanol and the CYP2C9*2 DNA capture probe in a self-assembled monolayer connected to screen-printed gold electrodes. Two independent hybridization events of the CYP2C9*2 allele were designed using complementary fluorescein-labeled DNA signaling to improve selectivity and avoid secondary structures. Three human samples with the homozygous variant (G/G) and non-variant (A/A) and heterozygous (G/A) genotypes were amplified by PCR and then applied to the developed genosensor. Results: Chronoamperometry measurements were performed for both polymorphic probes. A calibration curve in the 0.25 to 2.50 nM (LOD of 13 pM) and another in the 0.15 to 5.00 nM range (LOD of 22.6 pM) were obtained for the homozygous non-variant and variant probes, respectively. This innovative tool was capable of identifying the hybridization reaction between two complementary strands of immobilized DNA, representing a genotyping alternative to the classical PCR methodology. Conclusions: The developed electrochemical DNA-based sensor was able to discriminate two synthetic SNP target sequences (Target-A and Target-G) and detect, with specificity, the three patients’ genotypes (G/G, G/A, and A/A). This tool is therefore a promising, sensitive, and cost-effective analytical way to determine and discriminate an individual’s genotype and predict the appropriate warfarin dose.

## 1. Introduction

Personalized medicine is a scientific area that focuses on an individual’s unique and differentiated characteristics at a molecular, physiological, behavioral, and environmental level to minimize adverse drug reactions and clinical complications and, ultimately, improve the individual’s treatment outcome [1]. This new approach has been validated and implemented using developing tools, such as DNA sequencing, tomography procedures and artificial intelligence, machine learning, and proteomics, which have confirmed the existence of such inter-individual variations in several diseases [2,3]. Thus, everyone should be offered therapeutic interventions adapted and adjusted to their differentiating and specific genetic characteristics [1]. In a therapeutic context, pharmacogenomics is the scientific area closely related to personalized medicine, as it evaluates the influence of the patient’s genetic variations on their drug responses and treatment outcomes; i.e., pharmacogenetics relates a patient’s gene expression, to mutations or polymorphisms, like single-nucleotide polymorphisms (SNPs), or to the metabolism, efficacy, dose, suitability, or toxicity of a drug [4,5]. Briefly, pharmacogenomics aims to personalize an individual’s or ethnic group’s medical treatment by analyzing how their genome affects their response to drug doses of certain therapeutic compounds [4,5,6].

Cytochrome P450 (CYP) enzymes are implied in the biotransformation of 70% to 80% of all clinical drugs and are codified by CYP isoforms, which present genetic polymorphisms that can affect the enzyme’s expression and/or metabolic activity [7]. Among them, the cytochrome *P450-2C9* gene (*CYP2C9*), which encodes the CYP2C9 enzyme, is amply expressed and related to the metabolic rate of almost 15% of medications, including xenobiotics, such as warfarin—an anticoagulant drug commonly prescribed for the treatment and/or prevention of cardiovascular diseases [6,7]. Nonetheless, warfarin has a narrow medicinal window that severely affects a patient’s drug dose response. Hence, if the indicated dose for a patient is not appropriate, then negative reactions and/or complications may occur.

For instance, if the INR is higher than the therapeutic index, then the patient has a greater probability of hemorrhage, whereas patients with a lower INR value present an increased risk of suffering from thromboembolisms and/or strokes [6,8,9]. So, the identification of SNPs in the *CY2C9* gene, like the CYP2C9*2 (rs1799853) SNP, is essential to avoid potentially lethal health complications [10,11]. The identification of patients’ CYP2C9*2 genotype (rs1799853) SNP is especially important, to distinguish warfarin metabolizing status [12]. In this sense, it is important to develop analytical tools capable of genotyping these patients to determine their CYP2C9*2 genotype before prescribing a patient warfarin.

The standard analytical techniques for DNA genotyping and SNP detection are polymerase chain reaction (PCR) and real-time PCR (RT-PCR) [6,9,13,14], which are mainly based on detecting single-pair mismatches in ssDNA, distinguishing two alleles at a locus that differ by one nucleotide position, enzymatic difference cleavage, oligonucleotide covalent bonds, and single-strand conformation polymorphism determination. However, the fundamental issue with hybridization stability is its specificity, as the influence of each base mispair’s double-stranded structural stability is limited. In homogeneous hybridization, TaqMan probes are employed to detect fluorescence during RT-PCR SNP genotyping. Nevertheless, the expensive cost of apparatus and probes is the primary drawback to this analytical approach. On the other hand, due to their high throughput and affordability, DNA microarrays are the most frequently used tools for SNP detection. They are constrained by laborious processes and protracted operating times. Moreover, SNPs can also be reliably and quickly distinguished by DNA sequencing. Still, the procedure is quite costly, particularly when there are a lot of samples. For many years, the main obstacles have concerned sample specificity and analytical equipment costs.

Therefore, even if these tests are reasonable and precise, they are costly and time-consuming and require skilled specialists, restricting the ability of developing nations to genotype their citizens [9,12,15]. Hence, efforts have been made to develop new low-cost genotyping methods that generate faster and easier data to implement this screening process in public healthcare systems [6,13].

For example, to distinguish SNPs from vitamin K epoxide reductase complex subunit 1 (*VKORC1*) and the CYP2C9*2 and *3 alleles from actual DNA sequences produced by an allele-specific ligation isothermal and recombinase polymerase amplification, Lázaro et al. [16] used a multiplexed hybridization test established using Blu-Ray technology [16]. Likewise, Huang et al. [17] performed electrochemical readings with a sandwich layout DNA sensor composed of a thiol capture and a ferrocene-labeled signal to ascertain the genotype of patients for the *VKORC1* and CYP2C9*2 and CYP2C9*3 genes. A multi-PCR technique was also used to extract and amplify the genomic DNA of the volunteers [17]. Furthermore, in previous work, our group developed a DNA-based sensor to identify CYP2C9*3 SNPs with positive results [6].

Therefore, electrochemical DNA-based sensors are an excellent polymorphism genotyping alternative to classical PCR methodologies since these devices represent a promising and cost-effective analytical tool with the ability to detect the reaction between two complementary DNA sequences and, consequently, discriminate an individual’s genotype and predict the appropriate warfarin dose. So, similarly to our previous work [6], in this study, we aimed to report the development and optimization of a DNA-based sensor using an electrochemical detector for the detection of the non-variant homozygous (G/G), heterozygous (G/A), and homozygous variant (A/A) genotypes of the CYP2C9*2 SNP. Compared to previous research, this study has a higher sensitivity, presenting a detection limit of 13 pM, allowing us to identify SNPs with less DNA. Additionally, the measurement of the distinct electrochemical signals obtained for the three genotypes (G/G, G/A, and A/A) was the detection principle employed to develop this DNA-based sensor.

## 2. Materials and Methods

### 2.1. Equipment and Electrodes

Chronoamperometric readings were carried out on a Autolab potenciostat (Metrohm) controlled by NOVA 1.11.2 software. SPGEs from the DropSens (Spain) model C223AT were used as transducers in combination with a DRP-CAC73499 cable connector. Student’s *t*-test was used to calculate statistical significance, and a *p*-value < 0.05 was considered statistically significant. A Nanodrop spectrophotometer was used to quantify purified ssDNA; a centrifuge and vortex were also employed to perform this assay.

### 2.2. Chemicals, Samples, and Solutions

**Scheme 1:**
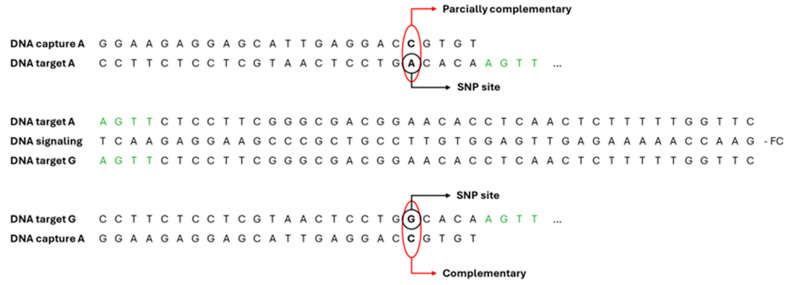
Complementarity of the DNA probes.

The chemicals used to perform this work were of the highest analytical grade. From Sigma-Aldrich, we purchased the saline buffer (SSPE 20x concentrate) and the enzymatic substrate (tetramethylbenzidine—TMB). The SPGE surface blockers (6- mercapto-1-hexanol—MCH) were obtained from Thermofisher, the antibody anti-fluorescein peroxidase (anti-FITC-POD) were obtained from Roch Diagnostics, and the lyophilized DNA salt sequence (Table 1) was obtained from Eurogentec. The electrochemical experimental methodology used for this study implies the immobilization of a DNA capture probe that targets both the fully complementary wild-type DNA target sequence (DNA-TG) and the partially complementary target probe (DNA-TA). Scheme 1 and Table 1 demonstrate the difference between both sequences.

Biological samples were obtained from the DNA bank of the population of Piauí, Brazil, which is part of the Piauí AIM Project of the Parnaíba Delta Federal University in Parnaíba, PI, Brazil. Details about the solution preparation are given in the Supplementary Materials.

### 2.3. Electrochemical DNA-Based Sensor Design

**Scheme 2:**
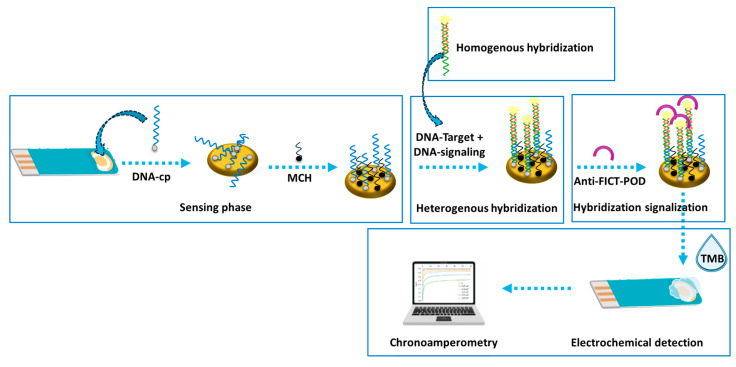
Design of the electrochemical DNA-based sensor.

To construct the electrochemical genosensor, several experimental steps were per-formed (Scheme 2): (a) SPGE cleaning; (b) the formation of a bilayer (ssDNA capture probe and MCH) on the SPGE surface; (c) the promotion of DNA hybridization using a sandwich format; and (d) chronoamperometric measuring. Details about all the protocols used and the preparation of the electrochemical genosensor are described in detail in the Supplementary Materials.

## 3. Results

### 3.1. Bioinformatic Tools to Select DNA Probes

To create an SNP-specific electrochemical genosensor, specific DNA oligonucleotide sequences capable of distinguishing the CYP2C9*2 SNP genetic variation region are necessary. Therefore, two 71 bp oligonucleotide sequences, DNA-Target G (DNA-TG) corresponding to the wild type genetic allele, and the genetic variant DNA-Target A (DNA-TA), were selected and designed. Afterwards, these two complementary sequences were sliced in silico to generate two smaller DNA fragments: a 46 bp DNA signaling probe and a 25 bp DNA capture probe, which were fully complementary with the Target-G probe (Table 1 and Scheme 1), and their energies were evaluated in order to minimize the formation of secondary structures that could hinder the hybridization process.

### 3.2. Optimization of the Experimental Parameters

To construct an analytical tool able of distinguishing the three CYP2C9*2 DNA sequences, all the trials were performed using both the DNA targets (TA and TG) with the non-variant DNA capture probe. Most of the experimental features, e.g., the concentration of the DNA capture and DNA signaling probes, the incubation time of the DNA signaling and the homogeneous and heterogenous hybridization steps, and the concentration and incubation time of the antibody and spacer involved in the DNA-based sensors, were adjusted (Table 2).

The optimizations of the experimental variables were performed according to three selection criteria, depending on the results obtained. The first condition, and the most valued one in the optimization process, was the largest ratio between the chronoamperometric currents read at −0.1 V for the control assay (blank, B) and the synthetic DNA target concentration (signal, S), designated the signal-to-blank ratio, S/B. The other factors taken into consideration were the current intensity of the electrochemical signals, i.e., the current intensity of the tested parameters, and the difference in the intensity between the DNA-TG, the DNA sequence complementary to the DNA capture (capture C) probe, and the partially complementary DNA-TA probe (TG/TA ratio).

**Figure 1:**
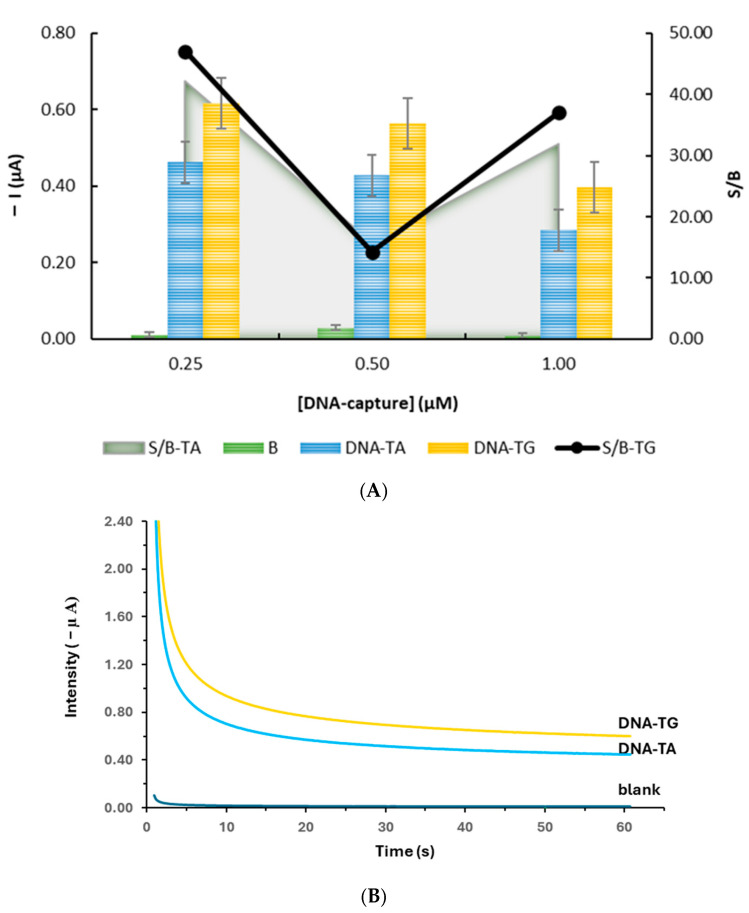
(**A**) Chronoamperometric currents when studying the impact of the DNA capture C probe concentration. Error bars estimate the standard deviation of three replicates (*p* < 0.05, paired *t*-test). (**B**) Chronoamperograms obtained for TA and TG.

Firstly, we studied the effect of the DNA capture probe concentrations on the chronoamperometric currents, and DNA capture concentrations ranging from 0.25 to 1.00 μM were immobilized on the SPGE working electrode surface (Figure 1).

By analyzing Figure 1, it is possible to verify that the highest S/B ratio value for both the DNA target probes was observed for SPGE immobilized with a DNA capture C probe concentration of 0.25 μM (S/B = 47.03 for TG and 42.23 for TA), followed by a concentration of 1.00 μM. The SPGEs immobilized with 0.50 μM of the DNA capture sequence registered the lowest S/B ratio, with values of 14.25 and 15.66 for non-variant TG and SNP variant TA probes, respectively. Nevertheless, as observed in Figure 1A, the increase in the DNA capture concentration led to a decrease in the chronoamperometric readings. Accordingly, the maximum cathodic electrochemical current intensity (Ic) was also acquired when 0.25 μM DNA capture C was utilized (Ic = 0.62 µA and 0.46 µA for TG and TA, respectively), followed by the 0.50 μM and 1.00 μM concentrations. Figure 1B presents the chronoamperograms obtained when testing 0.25 μM DNAcp.

**Figure 2:**
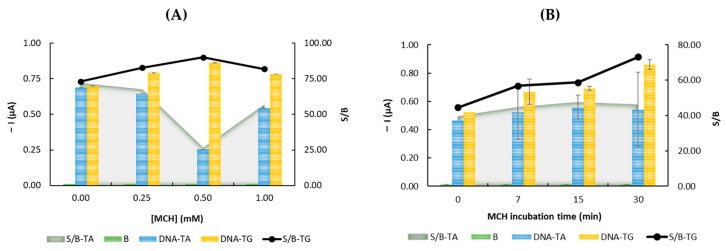
Effect of the MCH spacer (**A**) concentration and (**B**) incubation time on the chronoamperometric current. Error bars estimate the standard deviation of three replicates (*p* < 0.05, paired *t*-test).

The MCH concentration and its incubation time comprised the second parameter optimized. Based on the literature, a higher Ic can be achieved if DNA sequences, immobilized on the SPGE, are linearly orientated as a self-assembled monolayer, since the presence of orientated monolayers decreases non-specific bindings and provides more stability for the DNA sequence [6,17]. So, different concentrations of MCH, ranging from 0.00 to 1.00 µM (Figure 2A), over a 0; 7; 15; and 30 min incubation period (Figure 2B), were tested.

For the MCH concentration (Figure 2A), the highest S/B ratio, current intensity, and TA/TG ratio for the wild-type TG probe were achieved when the SPGE was immobilized with 0.50 µM of MCH (S/B = 90.13, Ic = 0.86 µA and TA/TG = 3.14). On the other hand, the SNP TA sequence registered the highest S/B ratio when no spacer was applied to the system (S/B ratio at 0.00 mM = 71.97).

Additionally, in the absence of MCH (0.00 µM), both the TA and TG probes registered very similar and elevated S/B ratios (71.97 and 73.18 for TA and TG, respectively) and Ic (0.69 µA for the partially complementary probe and 0.70 µA for the fully complementary sequence). Moreover, for TG, the 0.25 µM and 1.00 µM concentrations also achieved similar S/B ratios and current intensities (S/B ratio of 82.75 with an Ic of 0.79 µA and S/B ratio of 81.85 with a 0.78 µA for the 0.25 µM and 1.00 µM concentrations, respectively).

As for the MCH incubation time, the best Ic (0.86 µA) and S/B ratio (73.14) values for the fully complementary TG sequence were recorded after a 30 min incubation period (Figure 2B). Moreover, the incubation time of 30 min was also the analyzed time that presented the largest TA/TG ratio (TA/TG = 1.59). For the TG sequence, the increase in incubation time resulted in the intensification of both the S/B ratio and Ic; however, in the partially complementary TA sequence, there was no significant variation in their S/B ratio and Ic over time. The most prominent difference could be observed when no MCH was applied; i.e., for TA, the lowest Ic and S/B ratio was calculated in the absence of MCH, which recorded an Ic and S/B ratio of 0.42 µA and 39.46, respectively.

Greater time intervals were not tested, as it has been proven that, with MCH, incubations periods over 30 min can negatively impact the stability of the DNA on the electrode’s surface, leading to a diminished electrochemical response [6]. Therefore, the following optimizations were carried out using an MCH concentration of 0.50 µM for 30 min.

**Scheme 3:**
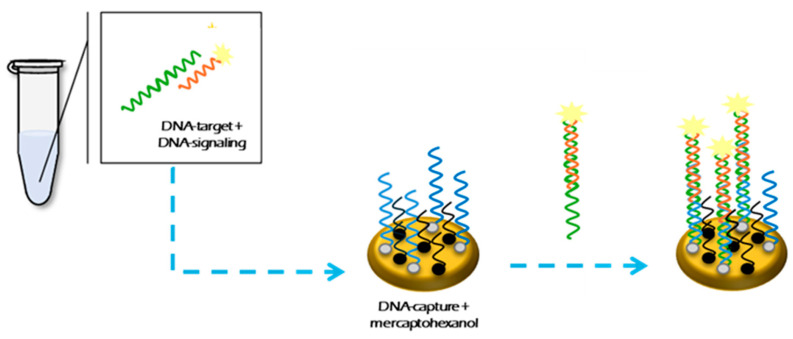
Homogeneous and heterogeneous hybridization process.

A sandwich hybridization format was utilized. This approach is advantageous for this work because the two independent hybridization events enhance the assay’s overall selectivity. In this process, homogeneous hybridization occurs due to the partial hybridization between the DNA target and DNA signaling. Meanwhile, heterogeneous hybridization takes place when the target–signaling duplex interacts with the capture probes immobilized on the SPGE (Scheme 3). So, prior to the complete hybridization of the wild-type and SNP target sequences, they must hybridize spontaneously with the DNA signaling probes (homogeneous hybridization reaction).

**Figure 3:**
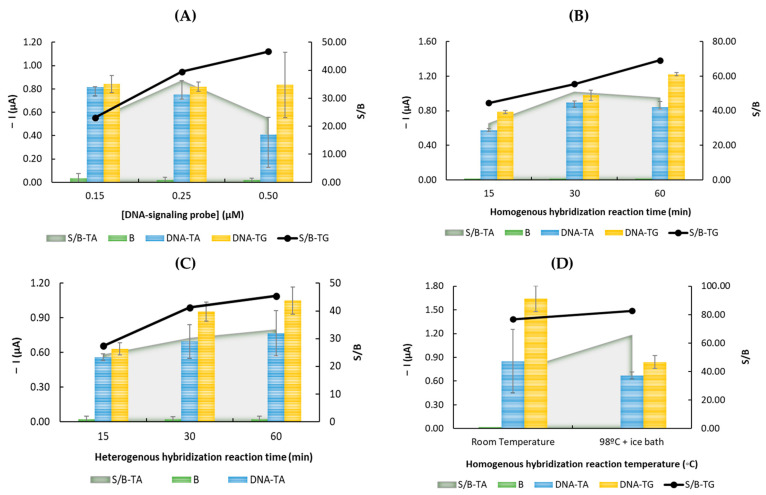
Chronoamperometric currents obtained when studying the (**A**) concentration of the DNA signaling probe, incubation time of the (**B**) homogeneous and (**C**) heterogeneous hybridization reactions, and (**D**) hybridization temperature. Error bars estimate the standard deviation of three replicates (*p* < 0.05 paired *t*-test).

The influence on the chronoamperometric response of the DNA signaling probe concentration (Figure 3A) and the incubation time on the homogeneous (Figure 3B) and heterogeneous (Figure 3C) hybridization reactions, as well as the hybridization temperature, (Figure 3D) was evaluated.

As observed in Figure 3A, increasing the concentration of the DNA signaling probe translated into a higher S/B ratio for the non-variant sequence (T_G_). Therefore, the best and highest S/B ratio for T_G_ (S/B = 46.86) was found when 0.50 µM of the DNA signaling probe was used, followed by the 0.25 µM (S/B = 26.67) and 0.15 µM (S/B = 22.35) concentrations. Nonetheless, the I_c_ for the all three concentrations was relatively similar, with the best I_c_ registered for the 0.15 µM DNA signaling concentration (I_c_ = 0.842 µA for T_G_), followed by 0.50 µM (I_c_ = 0.839 µA).

On the other hand, the maximum S/B ratio for the SNP variant T_A_ probe was attained with the 0.25 µM concentration (S/B = 36.27). However, the greatest I_c_ of all T_A_ assays (I_c_ = 0.82 µA) was recorded with the 0.15 µM concentration. Nevertheless, as 0.50 µM represented the appropriate S/B ratio for the completely complementary probe, as well as the highest T_G_/T_A_ ratio (2.05) and the second highest I_c_, this concentration was selected for all further optimizations.

As for the homogeneous and heterogeneous hybridizations (Figure 3B and Figure 3C, respectively) three distinct times, 15, 30, and 60 min, were studied to determine the influence of the incubation period on the electrochemical genosensor. Analyzing the results from both hybridization steps, it is evident that prolonged incubation periods provided higher S/B ratios, as well as higher I_c_ for the non-variant T_G_ sequence. The best S/B value for the homogeneous and heterogeneous hybridizations were achieved after the 60 min incubation period, with an S/B ratio of 69.18 and 45.38 for the homogeneous (Figure 3B) and heterogeneous (Figure 3C) reactions, respectively. Correspondingly, the best S/B ratio for the heterogeneous reaction of the variant SNP T_A_ probe was recorded after 60 min (Figure 3C). However, for the homogeneous reaction, the highest S/B ratio for T_A_ was registered after a 30 min interval (Figure 3B).

Finally, for the hybridization temperature (Figure 3D), two temperature conditions were analyzed: one at room temperature (around 25 ± 2 °C) and the other composed of a denaturation at 98 °C followed by an ice bath to produce a thermal shock. This denaturation process is normally applied when working with real DNA sequences to help separate the DNA’s double helix structure [6].

The best S/B ratios for both T_G_ (S/B = 82.63) and T_A_ (S/B = 65.66) were observed when the oligonucleotide probes were subjected to the thermal shock process.

Nonetheless, the highest I_c_ and T_G_/T_A_ ratios for the T_G_ sequences were achieved via the room temperature assay; i.e., at room temperature, an I_c_ of 1.64 µA and a T_G_/T_A_ ratio of 1.93 were obtained. Meanwhile, the other condition presented an I_c_ of 0.84 µA (nearly half of that for the previous condition) and a T_G_/T_A_ ratio of 1.26 for the wild-type oligonucleotide. Likewise, the altered T_A_ sequence also registered a low intensity, with an I_c_ of 0.67 µA. So, in comparison to T_G_, the only good response for the variant T_A_ was its S/B ratio, which only represents a 7.13% difference between the two. Therefore, all future optimizations and studies will remain at room temperature.

**Figure 4:**
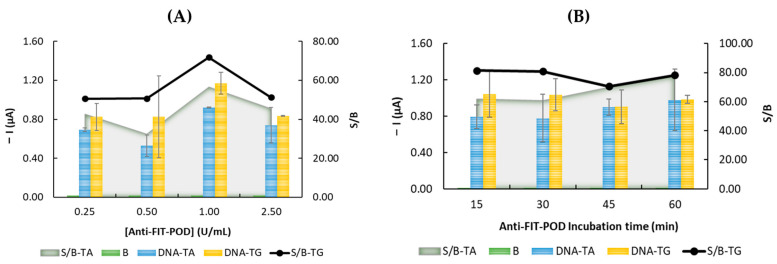
Chronoamperometric currents attained when studying the (**A**) concentration and (**B**) incubation time of the anti-FICT-POD enzymes. Error bars estimate the standard deviation of three to ten replicates (*p* < 0.05 paired *t*-test).

The final optimization step involved adjusting the amount and incubation time of the anti-FICT-POD enzymes. These enzymes are integrated into the fluorescein-labeled dsDNA. Consequently, the TMB/H_2_O_2_ substrate is introduced subsequently to the hybridization reaction s, and the number of POD enzymes is directly correlated with the number of hybridized sequences on the SPGE. To evaluate their impact on the performance of DNA-based sensors, anti-FICT-POD enzyme concentrations ranging from 0.25 to 2.50 U/mL (Figure 4A) were incubated on the electrode’s surface for varying durations, ranging from 15 to 45 min (Figure 4B).

According to the results illustrated in Figure 4A, it is clear that, for both sequences, the highest S/B ratios (71.83 for non-variant TG and 56.70 for the partially complementary TA sequence), as well as the highest Ic values (1.17 and 0.92 for TG and TA, respectively), were found when 1.00 U/mL of the anti-FITC-POD was applied after the hybridization reaction. As for the 0.25, 0.50, and 2.50 U/mL concentrations, all presented a similar S/B ratio for the TG probe (50.58, 50.69, and 51.28, respectively); however, for the variant TA sequence, the 0.50 U/mL concentration presented the lowest S/B ratio out of all assays.

However, for the anti-FITC-POD incubation time, the maximum S/B ratio (S/B = 81.50) and Ic (1.04 µA) for TG were achieved when the affinity reaction between the antibody and the dsDNA was performed for 15 min (Figure 4B), followed by the 30 min incubation period. Nevertheless, for the 45 and 60 min tests, there was no clear difference between the TG and TA sequences; i.e., both assays produced very similar S/B ratios and Ic values. Table 2 indicates the analytical features used in this work.

### 3.3. Analytical Performance

**Figure 5:**
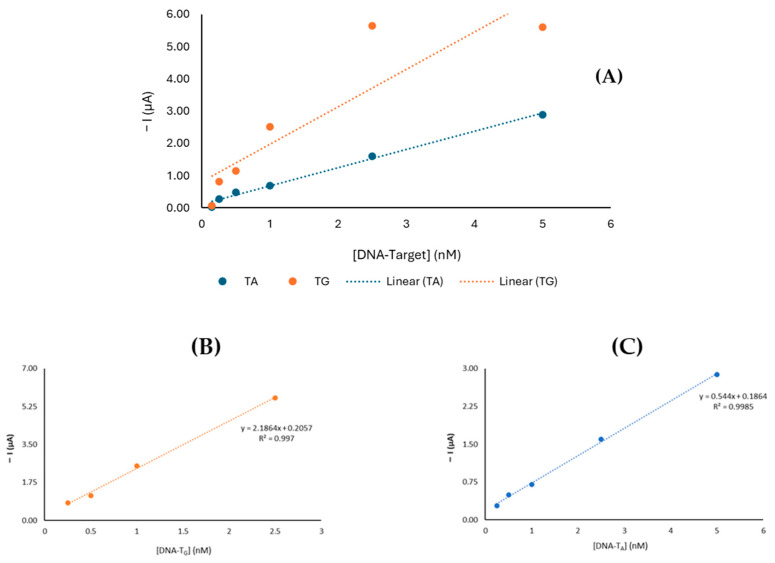
(**A**) Chronoamperometric responses for the blank subtracted current intensities and (**B**) calibration curve of the non-variant DNA-Target-G and (**C**) DNA-Target-A single-nucleotide polymorphism concentrations.

**Figure 6:**
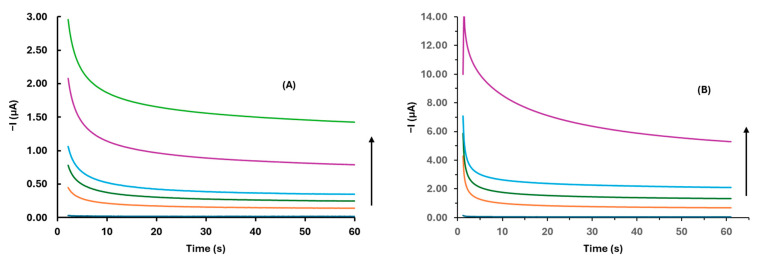
Chronoamperometric currents for (**A**) DNA-TA concentrations of 0.25; 0.5; 1.0; 2.5; and 5.0 nM and (**B**) DNA-TG concentrations of 0.25; 0.5; 1.0; and 2.5 nM.

With the selected analytical features (Table 2), the analytical characteristics of the DNA-based sensor were evaluated by chronoamperometry, applying increasing concentrations ranging from 0.15 to 5.00 nM (Figure 5A). A linear correlation (r^2^ = 0.997) between the chronoamperometric current and the DNA-TG concentration was obtained from 0.25 to 2.50 nM, with a slope and intercept value of 2.19 ± 0.11 (µA/nM) and 0.21 ± 0.09 (μA) (Figure 5B). On the other hand, the DNA-TA attained a linear relationship of 0.999 (r^2^ = 0.999), with a slope and intercept value of 0.54 ± 0.03 (µA/nM) and 0.19 ± 0.02 (μA) from the 0.15 to 5.00 nM range (Figure 5C). Figure 6 presents an example of the chronoamperograms obtained during this study. The limit of detection (LOD) and the limit of quantification (LOQ) were also calculated for each probe; the wild-type DNA-T_G_ probe attained an LOD of 13.03 pM and LOQ of 43.43 pM, while the T_A_ SNP sequence registered an LOD of 22.58 and an LOQ of 75.28 pM. Likewise, Huang et al. [17] conducted electrochemical experiments on a sandwich assay DNA sensor utilizing a capture modified with a thiol functional group and a label composed of ferrocene to evaluate the effect of *VKORC1* and *CYP2C9* genotypes on warfarin dosage. Although the quickest method (0.5 h) was developed by Huang et al. [17], the present work presents a higher accuracy in differentiating between the three genotypes.

### 3.4. Biological DNA Samples: Evaluation by an Electrochemical DNA-Based Sensor

The developed DNA-based sensor was used to identify the existence or the non-existence of SNPs in human blood samples. For this, blood was collected and then, using specific extraction kits, the genomic DNA was extracted and purified. Then, the developed electrochemical DNA-based sensor was applied to analyze the DNA collected from the DNA bank of Piauí AIM Project. Three samples from the genetic bank, equivalent to the homozygous wild-type G/G, heterozygous G/A, and homozygous variant SNP A/A genotypes, were selected and amplified by PCR.

Firstly, the amplified fragments were subjected to a denaturation process performed by warming these DNA sequences to 98 ± 1 °C for 5 min, followed by another 5 min in an ice bath. Then, 1 nM of the denatured PCR sample was mixed, at room temperature, with the DNA signaling probe to generate a hybridization reaction, and the chronoamperometric currents were registered.

**Figure 7:**
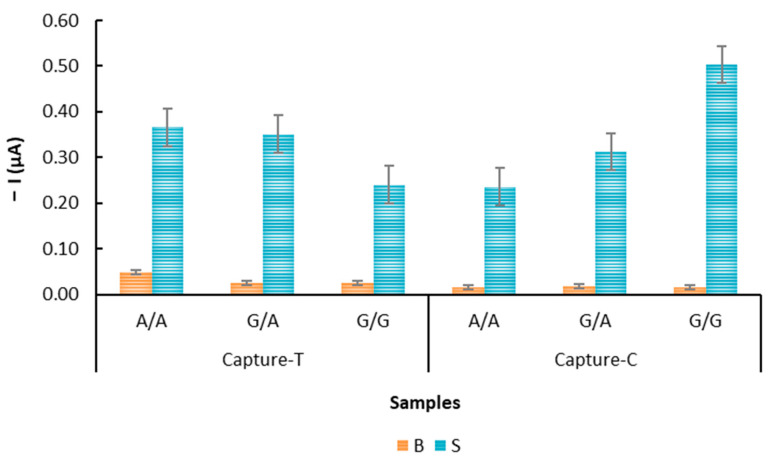
Comparison of the chronoamperometric responses detected after the hybridization of the homozygous single-nucleotide polymorphism variant A/A, heterozygous G/A, and wild-type homozygous G/G genotypes to the non-variant DNA capture C and DNA capture T variant probes. Error bars estimate the standard deviation of three replicates (*p* < 0.05 paired *t*-test).

Figure 7 displays the chronoamperometric currents obtained using amplified genomic genotyped DNA and synthetic complementary probes (DNA capture and DNA signaling). To further test the selectivity of the developed DNA-based sensor, two DNA capture probes—one complementary to the wild-type T_G_ sequence, i.e., the DNA capture-C probe, and another complementary to the SNP variant T_A_ sequence, i.e., the DNA capture T probe—were evaluated.

As observed in Figure 7, each fully complementary genotype presents the highest value for their corresponding DNA capture probe, and the sample with the highest Ic and S/B ratio was the A/A genotype, followed by the heterogeneous G/A sample and finally the homozygous G/G genotype, while for those immobilized with the DNA capture C sequences, the homozygous G/G genotype produced the highest S/B ratios and Ic values, followed by the heterozygous G/A genotype and the homozygous A/A sample, thus proving the developed sensor selectivity.

The best S/B ratio (33.12) and Ic (0.50 µA) were obtained with the G/G genotype when hybridized with the DNA capture C probe. However, contrary to what was expected, the A/A genotype/DNA capture T hybridization presented the lowest S/B ratio (7.48) of all assays. This result can be explained by the Ic value of its blank signal, since the A/A genotype/DNA capture T assay registered the highest blank signal (blank Ic = 0.05 µA).

On the other hand, the heterozygous G/A genotype registered Ic in the same range when reacted with the DNA capture T and capture-C; the G/A sample obtained Ic values of 3.51 µA and 3.12 µA when reacted with the DNA capture T and capture C, respectively.

These findings can be matched to previous work from our group in which a DNA-based sensor was built for the detection of CYP2C9*3 polymorphisms [6]. Compared to the CYP2C9*3 DNA-based sensor, the electrochemical detection of the polymorphisms in the CYP2C9*2 variation proved to be more challenging. This sensor not only presented lower electrochemical signals but also presented much closer non-variant/variant ratios. Nonetheless, there is a significant difference in the current intensity between the wild-type and SNP allele needed to determine a patient’s genotype with the constructed DNA-based sensor. To our knowledge, there are no other studies where an electrochemical DNA-based sensor based on the sandwich-format hybridization identification of SNP in the CYP2C9*2 genetic variation was developed.

## 4. Conclusions

An innovative tool based on an electrochemical DNA-based sensor able to discriminate against two polymorphic synthetic DNA probes with sensitivity and selectivity has been developed. To improve the SNP detection capability of the DNA-based sensor, optimizations of the various experimental analytical variables were performed. Thus, the construction of this tool with improved characteristics for the detection of CYP2C9*2 polymorphism was achieved.

The sensitivity of the DNA-based sensor was obtained through the DNA capture and mercaptohexanol spacer SAM formation on the SPGE. Another important aspect was the sandwich-format hybridization, which increased the selectivity of the DNA-based sensor. The detection of electrochemical currents was possible through the enzymatic reaction catalyzed by the POD enzyme, measured by the chronoamperometry technique.

The validation of the DNA-based sensor, as well as its selectivity and sensitivity, were confirmed by detection and amplified genomic DNA from the three genotypes (A/A, G/A, and G/G) with two different DNA capture sequences. This tool therefore represents a promising and cost-effective analytical way to determine and discriminate an individual’s genotype and predict the appropriate warfarin dose.

## Figures and Tables

**Table 1:** Oligonucleotide sequences. Letters in bold represent the SNP sites.

Oligonucleotides	Sequence 5′ → 3′	**Bp**
DNA capture	SHC_6_OH–GGA-A-GA-GGA-GCA-TTG-AGG-AC**C**-GTG-T	25
DNA signaling	TCA-AGA-GGA-AG**C**-CCG-CTG-CCT-TGT-GGA-GGA-GTT-GAG-AAA-AAC-CAA-G–FC	46
DNA target A	CTT-GGT-TTT-TCT-CAA-CTC-CTC-CAC-AAG-GCA-GCG-GGC-TTC-CTC-TTG-AAC-AC**A**-GTC-CTC-AAT-GCT-CCT-CTT-CC	71
DNA target G	CTT-GGT-TTT-TCT-CAA-CTC-CTC-CAC-AAG-GCA-GCG-GGC-TTC-CTC-TTG-AAC-AC**G**-GTC-CTC-AAT-GCT-CCT-CTT-CC	71

**Table 2:** Analytical features used to optimize the construction of the electrochemical DNA-based sensor.

Variables	Tested Range	Selected Value
DNAcp concentration (μM)	0.25–1.00	0.25
MCH concentration (mM)	0.25–1.00	0.50
MCH incubation time (min)	7.5–30	30
Homogeneous hybridization incubation time (min)	15–45	30
DNA signaling concentration probe (μM)	0.13–0.50	0.50
Heterogeneous hybridization incubation time (min)	30–120	60
anti-FICT-POD enzyme concentration (U/mL)	0.50–2.50	1.00
anti-FICT-POD enzyme incubation time (min)	15–60	60

## Data Availability

No new data were created or analyzed in this study. Data sharing is not applicable to this article.

## Supplementary Materials

The following supporting information can be downloaded at: https://www.mdpi.com/article/10.3390/genes16040372/s1. Supplementary File S1: The protocols for the (i) solutions preparation, (ii) eletrochemical genosensor design and (iii) DNA samples [18].

## References

[1] Elemento O. (2020). The future of precision medicine: Towards a more predictive personalized medicine. Emerg. Top. Life Sci..

[2] Goetz L.H., Schork N.J. (2018). Personalized medicine: Motivation, challenges and progress. Fertil. Steril..

[3] Johnson K.B., Wei W.Q., Weeraratne D., Frisse M.E., Misulis K., Rhee K., Zhao J., Snowdon J.L. (2021). Precision Medicine, AI and the Future of Personalized Health Care. Clin. Transl. Sci..

[4] Valdes R., Yin D.T. (2016). Fundamentals of Pharmacogenetics in Personalized, Precision Medicine. Clin. Lab. Med..

[5] Dere W.H., Suto T.S. (2009). The role of pharmacogenetics and pharmacogenomics in improving translational medicine. Clin. Cases Miner. Bone Metab..

[6] Morais S.L., Magalhães J.M.C.S., Domingues V.F., Delerue-Matos C., Ramos-Jesus J., Ferreira-Fernandes H., Pinto G.R., Santos M., Barroso M.F. (2023). Development of an electrochemical DNA-based biosensor for the detection of the cardiovascular pharmacogenetic-altering SNP *CYP2C9*3*. Talanta.

[7] Zhang Q., Qi Y., Wang S., Zhao F., Zou L., Zhou Q., Geng P., Hong Y., Yang H., Luo Q. (2023). Identification and in vitro functional assessment of 10 CYP2C9 variants found in Chinese Han subjects. Front. Endocrinol..

[8] Panchenko E., Kropacheva E., Dobrovolsky A., Titaeva E., Zemlyanskaya O., Trofimov D., Galkina I., Lifshits G., Vereina N., Sinitsin S. (2020). CYP2C9 and VKORC1 genotyping for the quality of long-standing warfarin treatment in Russian patients. Pharmacogenomics J..

[9] Morais S.L., Magalhães J., Domingues V., Delerue-Matos C., Ramos-Jesus J., Ferreira-Fernandes H., Pinto G.R., Barroso M.F. Development of electrochemical genosensors applied to cardiovasular pharmacogenetics. Proceedings of the 1st International Electronic Conference on Chemical Sensors and Analytical Chemistry.

[10] Ong F.S., Deignan J.L., Kuo J.Z., Bernstein K.E., Rotter J.I., Grody W.W., Das K. (2012). Clinical utility of pharmacogenetic biomarkers in cardiovascular therapeutics: A challenge for clinical implementation. Pharmacogenomics.

[11] Karki R., Pandya D., Elston R.C., Ferlini C. (2015). Defining “mutation” and “polymorphism” in the era of personal genomics. BMC Med. Genom..

[12] Dean L., Pratt V.M., Scott S.A., Pirmohamed M., Esquivel B., Kattman B.L., Malheiro A.J. (2012). Warfarin Therapy and *VKORC1* and *CYP* Genotype. Medical Genetics Summaries.

[13] Abad-Valle P., Fernández-Abedul M.T., Costa-García A. (2007). DNA single-base mismatch study with an electrochemical enzymatic genosensor. Biosens. Bioelectron..

[14] Hosseinkhani Z., Sadeghalvad M., Norooznezhad F., Khodarahmi R., Fazilati M., Mahnam A., Fattahi A., Mansouri K. (2018). The effect of *CYP2C9*2*, *CYP2C9*3* and *VKORC1-1639 G>A* polymorphism in patients under warfarin therapy in city of Kermanshah. Res. Pharm. Sci..

[15] Liu H.Y., Hopping G.C., Vaidyanathan U., Ronquillo Y.C., Hoopes P.C., Moshirfar M. (2019). Polymerase Chain Reaction and Its Application in the Diagnosis of Infectious Keratitis. Med. Hypothesis Discov. Innov. Ophthalmol..

[16] Lázaro A., Yamanaka E.S., Maquieira Á., Tortajada-Genaro L.A. (2019). Allele-specific ligation and recombinase polymerase amplification for the detection of single nucleotide polymorphisms. Sens. Actuators B Chem..

[17] Huang T.-S., Zhang L., He Q., Li Y.-B., Dai Z.-L., Zheng J.-R., Cheng P.-Q., He, Y-S (2017). DNA sensors to assess the effect of *VKORC1* and *CYP2C9* gene polymorphisms on warfarin dose requirement in Chinese patients with atrial fibrillation. Australas. Phys. Eng. Sci. Med..

[18] Lopes T.R., Santos S., Ribeiro-dos-Santos Â., Resque R.L., Pinto G.R., Yoshioka F.K.N. (2014). Population Data of the 46 Insertion–Deletion (INDEL) Loci in Population in Piauí State, Northeastern Brazil. Forensic Sci. Int. Genet..

