# Supplementary material for: Warfarin Pharmacogenomics: Designing Electrochemical DNA-Based Sensors to Detect CYP2C9*2 Gene Variation
Source: Genes (Basel). 2025 Mar 24;16(4):372. doi: 10.3390/genes16040372 (PMC12027050; doi:10.3390/genes16040372)
Supplement: Supplementary file 1 [file genes-16-00372-s001.zip › genes-3488049-supplementary.pdf]

SUPPORTING INFORMATION

Warfarin Pharmacogenomics: Design of an Electrochemical DNA-Based Sensor for the Detection of CYP2C9*2 Gene Variation

Tiago Barbosa [1,2], Stephanie L. Morais [2], Eduarda Pereira [1,2], Júlia M. C. S. Magalhães [3], Valentina F. Domingues [2], Hygor Ferreira-Fernandes [4], Giovanny R. Pinto [5], Marlene Santos [1,6] and Maria Fátima Barroso [2,*]

**CONTENTS**	**PAGE**
Solutions preparation	2
Eletrochemical genosensor design	2
DNA samples	2
References	3

Solutions preparation

the saline buffer (SSPE 20x concentrate) was diluted to a concentration of 2x using Milli-Q ultrapure water obtained from a Millipore purification system.

The efficacy of the electrochemical genosensor depends on the specificity of the probes selected to detect CYP2CP*2 SNP. So, DNA sequences were specifically design using public databases (NCBI). Hence, two specific 71 bp target sequences; one with the adenine (ssDNA$\mathbf{T}_A$) and another with the guanine (ssDNA$\mathbf{T}_G$) SNP genetic variation were selected and designed. Every oligonucleotide stock solution (100 µM) was prepared with Milli-Q ultrapure water and stored at - 20°C, while the working oligonucleotides were prepared daily by diluting the desired concentration in the 2x SSPE buffer. The target's complementary probes capture and signalling probes were divided as a 25 bp and 46 bp sequence, respectively. The DNA-capture probe was functionalized with a thiol group at the 5′ end to enable its attachment onto the gold substrate, whereas the signalling probe was functionalized with a protein fluorescein – at its 3′ end. Theoretically, the capture and signalling probes will form a perfect and rigid duplex with the complementary target sequence.

Eletrochemical genosensor design

To construct the electrochemical genosensor, firstly the SPGEs were cleaning with ethanol, and ultrapure water, then drying with a nitrogen flow.

On the second step a bilayer was formed by the immobilization of the ssDNAcp and mercapto-1-hexanol (MCH) at the SPGE surface. For that, 3 µL of 1 µM ssDNAcp was added onto the working electrode and stored in a humidified Petri dish overnight. Then, the modified SPGE was rinsed twice with 2x SSPE buffer (to remove the weakly attached DNA probes) and 3 µL of MCH (1 µM) was added to the modified electrodes.

The DNA hybridization reaction was performed in a two-step hybridization. First, the homogenous hybridization occurred when a ssDNAsp (0.25 µM) binds to the ssDNA target in buffer solution, for 30 min. Then the heterogenous hybridization was promoted by adding the result of the homogenous hybridization onto the modified electrode (DNASPGEs). After 60 minutes, the total hybridization was carried out. Then, the electrodes were rinsed with 2X SSPE to remove any dense nonspecific DNA chains.

At the end, and to amplify the electrochemical signal it was added to the modified dsDNA-SPGEs an Anti-FIT-POD enzyme. Enzymatic amplification of the electrochemical signal was obtained through chronoamperometry through a TMB/H_2O_2 system. Enzymatic labelling using monovalent bonds provides an improvement in terms of detection limits, while at the same time introducing scaling selectivity. In the end, the genosensor was connected to a potentiostat and 40 µL of TMB/H_2O_2 substrate was added to cover the electrode for 1 min. Detection of the enzymatically oxidized product was made by chronoamperometry at – 0.1 V, for 60 seconds.

DNA samples

DNA samples of the patients were selected from the ancestry-informative marker (AIM) DNA bank of the population of Piauí, Brazil, which is part of the Piauí AIM Project by the Parnaíba Delta Federal University in Parnaíba, PI, Brazil. The procedure for extracting DNA and quantifying it was carried according to Lopes, T.R (2018) [1] The research project was granted authorization by the Research Ethics Committee of the Federal University of Piauí, Teresina, PI, Brazil, and it was carried out with the informed consent of all the participants.

Reference:

[1] Lopes, T.R.; Santos, S.; Ribeiro-dos-Santos, Â.; Resque, R.L.; Pinto, G.R.; Yoshioka, F.K.N. Population Data of the 46 Insertion–Deletion (INDEL) Loci in Population in Piauí State, Northeastern Brazil. Forensic Science International: Ge-netics 2014, 9, e13–e15, doi:10.1016/j.fsigen.2013.07.009.
